# Characterization of *Tg(Etv4-GFP)* and *Etv5*^*RFP*^ Reporter Lines in the Context of Fibroblast Growth Factor 10 Signaling During Mouse Embryonic Lung Development

**DOI:** 10.3389/fgene.2019.00178

**Published:** 2019-03-14

**Authors:** Matthew R. Jones, Arun Lingampally, Salma Dilai, Amit Shrestha, Barry Stripp, Francoise Helmbacher, Chengshui Chen, Cho-Ming Chao, Saverio Bellusci

**Affiliations:** ^1^Department of Pulmonary and Critical Care Medicine, The First Affiliated Hospital of Wenzhou Medical University, Wenzhou, China; ^2^Department of Internal Medicine II, Member of the German Lung Center, Cardio-Pulmonary Institute, University of Giessen Lung Center, Giessen, Germany; ^3^Department of Medicine, Cedars-Sinai Medical Center, Lung and Regenerative Medicine Institutes, Los Angeles, CA, United States; ^4^Aix Marseille University, CNRS, IBDM, UMR7288, Marseille, France; ^5^Department of General Pediatrics and Neonatology, University Children's Hospital Gießen, Justus-Liebig-University, Gießen, Germany; ^6^International Collaborative Center on Growth Factor Research, Life Science Institute, Wenzhou University-Wenzhou Medical University, Wenzhou, China

**Keywords:** ETV4, ETV5, FGF10, lung development, branching morphogenesis

## Abstract

Members of the PEA3 transcription factors are emerging as bone fide targets for fibroblast growth factor (FGF) signaling. Among them, ETV4 and ETV5 appear to mediate FGF10 signaling during early embryonic lung development. In this paper, recently obtained *Tg(Etv4-GFP)* and *Etv5*^*CreERT*2−*RFP*^ fluorescent reporter lines were generally characterized during early embryonic development and in the context of FGF10 signaling, in particular. We found that both *Tg(Etv4-GFP)* and *Etv5*^*CreERT*2−*RFP*^ were primarily expressed in the epithelium of the lung during embryonic development. However, the expression of *Etv5*^*CreERT*2−*RFP*^ was much higher than that of *Tg(Etv4-GFP)*, and continued to increase during development, whereas *Tg(Etv4-GFP)* decreased. The expression patterns of the surrogate fluorescent protein GFP and RFP for ETV4 and ETV5, respectively, agreed with known regions of FGF10 signaling in various developing organs, including the lung, where ETV4-GFP was seen primarily in the distal epithelium and to a lesser extent in the surrounding mesenchyme. As expected, ETV5-RFP was restricted to the lung epithelium, showing a decreasing expression pattern from distal buds to proximal conducting airways. FGF10 inhibition experiments confirmed that both *Etv4* and *Etv5* are downstream of FGF10 signaling. Finally, we also validated that both fluorescent reporters responded to FGF10 inhibition *in vitro*. In conclusion, these two reporter lines appear to be promising tools to monitor FGF10/FGFR2b signaling in early lung development. These tools will have to be further validated at later stages and in other organs of interest.

## Introduction

PEA3 transcription factors are a subfamily of the E26 transformation-specific (ETS) transcription factor family consisting of three members: ETV1 (also known as Er81), ETV4 (also known as PEA3), and ETV5 (also known as ERM) (reviewed by Sharrocks et al., 1997). Evidence from multiple studies has demonstrated that ETV4 and ETV5 are primary mediators of fibroblast growth factor (FGF) signaling via fibroblast growth factor receptor 2b (FGFR2b), and play overlapping roles in the patterning, morphogenesis, differentiation, and homeostasis of multiple organs and structures. For example, ETV4 and ETV5 have been shown to repress *Shh* expression in mouse limb buds, thus promoting bud outgrowth and proper anterior-posterior patterning (Mao et al., 2009; Zhang et al., 2009); studies in the mouse lung have shown that ETV4- and ETV5-mediated induction of *Shh* appear to regulate branching morphogenesis (Herriges et al., 2015); furthermore, ETV5 was shown to maintain alveolar type 2 (AT2) cell identity during mouse lung homeostasis and repair after injury, and has been implicated in lung tumorigenesis (Zhang et al., 2017); during development of the lacrimal gland in mice, it was demonstrated that PEA3 transcription factors control epithelial cell fate determination (Garg et al., 2018); in the kidney as well, research has shown that ETV4 and ETV5 play a wide range of functions within multiple signaling pathways, including FGF signaling, to regulate Wolffian duct and ureteric bud morphogenesis (Kuure et al., 2010); finally, a study in zebrafish has found that knocking down *Etv4* and *Etv5* resulted in embryonic abnormalities similar to a loss of FGF signaling, including cardiac, and left/right patterning defects (Znosko et al., 2010).

Lung organogenesis is a complex process involving a number of signaling pathways, and is divided into distinct stages comprising embryonic (E) and postnatal (P) development. In the mouse, these stages include the pseudoglandular stage (E9.5–16.5), the canalicular stage (E16.5–17.5), the saccular stage (E17.5–P5), and the alveolar stage (P5–P30) (reviewed in Chao et al., 2015). During each of the embryonic and postnatal stages, FGF signaling plays critical roles. For instance, during the pseudoglandular stage of lung development, the majority of the lung architecture is established via branching morphogenesis, and also most of the epithelial and mesenchymal cell types are formed (reviewed in El Agha and Bellusci, 2014). Disruption of FGFR2b signaling during this stage leads to morphological and differentiation defects (Bellusci et al., 1997b), while either *Fgf10-* or *Fgfr2b-*null embryos display complete lung agenesis (Sekine et al., 1999; De Moerlooze et al., 2000).

Another critical signaling pathway during pseudoglandular lung development is the sonic hedgehog (SHH) pathway. SHH is a negative regulator of *Fgf10* expression in the distal mesenchyme, and serves to limit the action of FGF10/FGFR2b signaling (Bellusci et al., 1997a). Recent studies propose that FGF10 acts on SHH via ETV4 and ETV5, creating an FGF10/ETV4/ETV5/SHH axis required for orchestrating proper branching morphogenesis (Herriges et al., 2015). It is unclear, however, how FGF10 acts on ETV4 and ETV5; whether it acts directly to regulate the expression of these transcription factors, for example, or whether these are regulated indirectly via another pathway.

To aid the study of the role of ETV4 and ETV5 during organogenesis, two transgenic reporter mice have recently been developed, *Tg(Etv4-GFP)* (Lamballe et al., 2011) and *Etv5*^*CreERT*2−*RFP*^ (also referred to as *Etv5*^*RFP*^). These lines report, via green and red fluorescent markers, the expression of *Etv4* and *Etv5*, respectively. Furthermore, *Etv5*^*CreERT*2−*RFP*^ can be used to drive inducible genetic recombination in *Etv5* positive cells, upon administration of tamoxifen. These lines can, therefore, potentially be used as invaluable research tools to study the role of ETV4 and ETV5 during the development of multiple organs, especially in the context of FGF signaling.

In this paper, we characterize these two reporter mouse lines during early embryogenesis (up to E16.5). We report the areas and organs of expression of ETV4-GFP and ETV5-RFP in the mouse embryo. We then focus on the expression of *Etv4* and *Etv5* in isolated embryonic lung mesenchyme and epithelium as well as the dynamic expression of the ETV4-GFP and ETV5-RFP protein during pseudoglandular development. Finally, various FGF10 inhibition experiments were carried out *in vitro* to determine the impact on the expression of ETV4-GFP and ETV5-RFP activity.

## Materials and Methods

### Animal Husbandry and Experimental Embryos

All animals were housed in a specific-pathogen-free (SPF) facility in accordance with local, state, and national laws. Timed pregnancies were set-up to obtain experimental embryos for analysis at the desired embryonic stage (from E9.5 to E18.5). Noon on the day of the vaginal plug was considered E0.5.

To obtain experimental and littermate control embryos, mice heterozygous for *Etv5*^*CreERT*2−*RFP*^ (*B6.Cg-Etv5*<*tm1(cre/ERT2)Brst*>), or hemizygous for *Tg(Etv4-GFP)* (*B6-Tg(Etv4/EGFP)4Fhel*) (Lamballe et al., 2011), were crossed with wild type mice. Embryos were harvested at the desired embryonic stage. Briefly, pregnant females were sacrificed with an overdose of pentobarbital sodium (dosage: 0.4 mg pentobarbital / g mouse weight), embryos removed, and washed in PBS for ~2 min. Organs were dissected under a stereoscope and positioned for imaging.

DNA was extracted from tails and limbs following basic lab procedures and prepared for PCR-based genotyping. The *Tg(Etv4-GFP)* gene was detected using the following primer sequences: Forward−5′-GGA ATC TTG GGC CTT GAG AAC AGC-3′; reverse−5′-CGC TGA ACT TGT GGC CGT TTA CG-3′. The cycling protocol was as follows: denaturation at 94°C for 3 min; 40 cycles of denaturation at 94°C for 15 s, annealing at 60°C for 30 s, and extension at 72°C for 1 min; finish with a final extension at 72°C for 5 min. The *Etv5*^*CreERT*2−*RFP*^ gene as well as the wild type *Etv5*, was detected using the following primer sequences: *Etv5*^*CreERT*2−*RFP*^ forward−5′-TCG ATG CAA CGA GTG ATG AG-3′; *Etv5*^*CreERT*2−*RFP*^ reverse−5′-TTC GGC TAT ACG TAA CAG GGT-3′; *Etv5* forward−5′-AAA GAG GAA CGC GGT CTG AG-3′; *Etv5* reverse−5′-CCA GCT GAG TCT CGT GTG AT-3′. The cycling protocol was as follows: denaturation at 95°C for 3 min; 30 cycles of denaturation at 94°C for 30 s, annealing at 60°C for 30 s, and elongation at 72°C for 80 s. Product bands were detected by capillary gel electrophoresis.

### FACS-based Isolation of Epithelial and Mesenchymal Cells

Lungs from embryonic mice were dissected at desired time points (E14.5, E16.5, and E18.5), and transferred to ice-cold Hank's Balanced Salt Solution (HBSS). Lungs were finely chopped with a sterile razor blade on a glass plate. The tissue was then added to a falcon tube and digested in 0.5% collagenase at 37°C for 45 min, with constant mixing. Single cell suspensions were made by successively flushing the samples through 18, 20, and 24 g grade needles and then filtering the samples through 70 and 40 μm nylon strainers (BD Biosciences). The cell suspensions were diluted with 5 ml HBSS and centrifuged at 12,000 rpm for 5 min. and the supernatant was discarded. The pellet was resuspended in 10 μl blocking buffer and the following antibodies were added: 488-CD31 (1:50); FITC-CD45 (1:50); and Apc Cy7 EpCam (Epithelial cell adhesion molecule) (1:50) (Biolegend), for 20 min at 4°C. The samples were washed 2x with 100 μl FACS buffer and centrifuged at 12,000 rpm for 5 min at 4°C. The supernatant was discarded. The pellet was resuspended in 100 μl FACS buffer.

Cell sorting and isolation was performed using the FACSAria™ III (BD Biosciences) cell sorter. Alveolar epithelial cells were identified as CD45^−ve^/CD31^−ve^/ Epcam^+ve^ and mesenchymal cells as CD45^−ve^/CD31^−ve^/ Epcam^−ve^. Cells were sorted through a flow chamber with a 100-μm nozzle tip under 25 psi sheath fluid pressure. Isolated cells were used for RNA isolation. As a main criterion for gating, we used the settings allowing to capture 98% of the cells in the isotype control and then we applied these gating conditions to the stained cells.

### RT-qPCR

Either FACS-isolated cells, or embryonic lungs were lysed in Qiazol lysis reagent, and total RNA isolated using a miRNeasy mini RNA extraction kit according to the manufacturer's instructions (Qiagen). Please note that from cell dissociation to RNA isolation, the process takes 3–4 h. Our results indicate that there is very little variability between samples from the same time point and same group (epithelium or mesenchyme).

Up to 1 μg RNA was reverse-transcribed using the QuantiTect reverse transcription kit (Qiagen). cDNA was used to specifically amplify the desired DNA sequence by quantitative PCR (qPCR). Primers were designed to amplify specific mature mRNAs using NCBI's primer-BLAST option (https://www.ncbi.nlm.nih.gov/tools/primer-blast/) (last accessed, 01-08-2018). Primers were further validated by PCR-based gel electrophoresis. The following primer sequences were used: *Etv4* (FWD-5′-CAGACTTCGCCTACGACTCA-3′; REV-5′- GCCATAACCCATCACTCCAT-3′); *Etv5* (FWD-5′-GTGGCCGCTCAGGAGTA-3′; REV-5′-GTGCTTCCTTCCAAAGTCTCCG CT-3′); *RFP* (FWD-5′-GCGTGATGAACTTCGAGGAC-3′; REV-5′-TTCACCTTG TAGATCAGCGTG-3′); *Hprt* (FWD-5′-CCTAAGATGAGCGCAAGTTGAA-3′; REV-5′-CCACAGGACTAGAACACCTGCTAA-3′).

qPCR reaction mixtures were set up using the PowerUp SYBR Green Master Mix kit following the manufacturer's instructions (Thermo Fisher). Samples were run with three technical replicates on a LightCycler 480II (Roche) using the following protocol: UDG activation at 50°C for 2 min; DNA polymerase activation at 95°C for 2 min; and 40 cycles of denaturation at 95°C for 15 s, annealing at 60°C for 15 s, and extension at 72°C for 1 min. To validate amplification specificity, a dissociation step was also included for each sample. Threshold cycles (Ct) were calculated and used for relative expression analyses, using mouse *Hprt* as the reference gene.

ΔCt and ΔΔCt values were calculated according to the following formulas:

$$\begin{array}{l} \Delta \text{Ct}={\text{Ct}}_{\text{Reference}}-{\text{Ct}}_{\text{geneof interest}} \end{array}$$

Note, this equation accounts for the fact that Ct is proportional to the –log of gene expression. ΔCt is therefore positively related to the expression of the gene of interest.

Expression relative to *Hprt* = 2^Δ*Ct*^

$$\begin{array}{l} \Delta \Delta {\text{Ct}}_{\text{Experimental}-\text{control}}=\text{ Mean}\Delta {\text{Ct}}_{\text{Experimental}}-\text{Mean}\Delta {\text{Ct}}_{\text{Control}} \end{array}$$

Unpaired two-tailed Student's *t*-tests were performed on the ΔCt values, which can be assumed to be normally distributed. Number of “n” form of graphical representation, and significance level is indicated either in the figures or in the figure legends.

### Lung Explant Culture for *in vitro* Experiments

Embryonic lungs used for *in vitro* experiments were obtained either from genetically modified embryos generated as described above, or from C57BL/6 wild-type embryos.

Embryonic lungs were dissected and cultured on 13 mm Whitman Track-Etch polycarbonate membranes, with 8.0 μm pores (Merck) positioned atop DMEM culture medium in a 24-well culture dish [medium contained: Dulbecco's Modified Eagle Medium (1x DMEM), supplemented with D-Glucose, L-Glutamine, HEPES, Pyruvate, and Phenol red (Gibco), 10% fetal bovine serum (FBS), 1% penicillin (10,000 units/ml)-streptomycin (10 mg/ml)]. Lungs were incubated at 5% CO_2_ and 37°C for ~45 min to allow them to settle. At the desired time, recombinant FGF7 (50 ng/ml), FGF10 (250 ng/ml), soluble FGFR2b (5 μg/ml) (R and D Systems), or anti-FGF10 blocking antibody (20 μg/ml) (Santa Cruz Biotechnology) was added to the experimental lungs (as described in Sakaue et al., 2002), while the vehicle was added to control samples. Lungs were incubated at 5% CO_2_ and 37°C for the duration of the experiment.

### *In vivo* Model to Inhibit FGFR2b Signaling

*In vivo* studies were conducted using an inducible dominant negative mouse model: *Rosa26*^*rtTA*/+^*; Tg(tetO-Fgfr2b)/*+ (*B6-Cg-Gt(ROSA)26Sor*^*tm*1.1(*rtTA, EGFP*)*Nagy*^ *Tg(tetO-Fgfr2b/lgh)1.3Jaw/sbel*). Doxycycline was administered via intraperitoneal injection to timed-pregnant females, as previously described (Danopoulos et al., 2013).

### Imaging Acquisition and Measurements

Brightfield images of lungs from *in vivo* and *in vitro* experiments were captured either on a Leica MZ 125 stereoscopic dissecting microscope using a Spot Insight 2.0 Mp Color Mosaic camera and Spot 4.5.9 imaging software, or were obtained from live imaging experiments using a Leica DM6000B inverted microscope, DFC 305FX camera, and Leica Application Suite Advanced Fluorescence imaging software. Fluorescence intensities were quantified using FIJI software (version 2.0.0-rc-68/1.52 g).

Branching was quantified by counting and averaging the distal tips of the left lobe of control and experimental samples at 0 and 24 h. These groups were analyzed using a two-factorial quasi-Poisson model, with the dispersion parameter estimated as 0.317. Differences were considered statistically significant at *p* < 0.05.

### Gene Expression Patterns

To assess the expression patterns of genes in early stage embryonic lungs (E14.5), the online database genepaint.org was used (last accessed 01-08-2018). Each of the genes significantly regulated in our *in vivo* studies was entered in genepaint. The whole embryo section displaying the clearest gene expression in the lung, along with a magnification of the lung itself, was chosen for the figures.

## Results and Discussion

### *Tg(Etv4-GFP)* Expression During Early Embryonic Development

**Figure 1:**
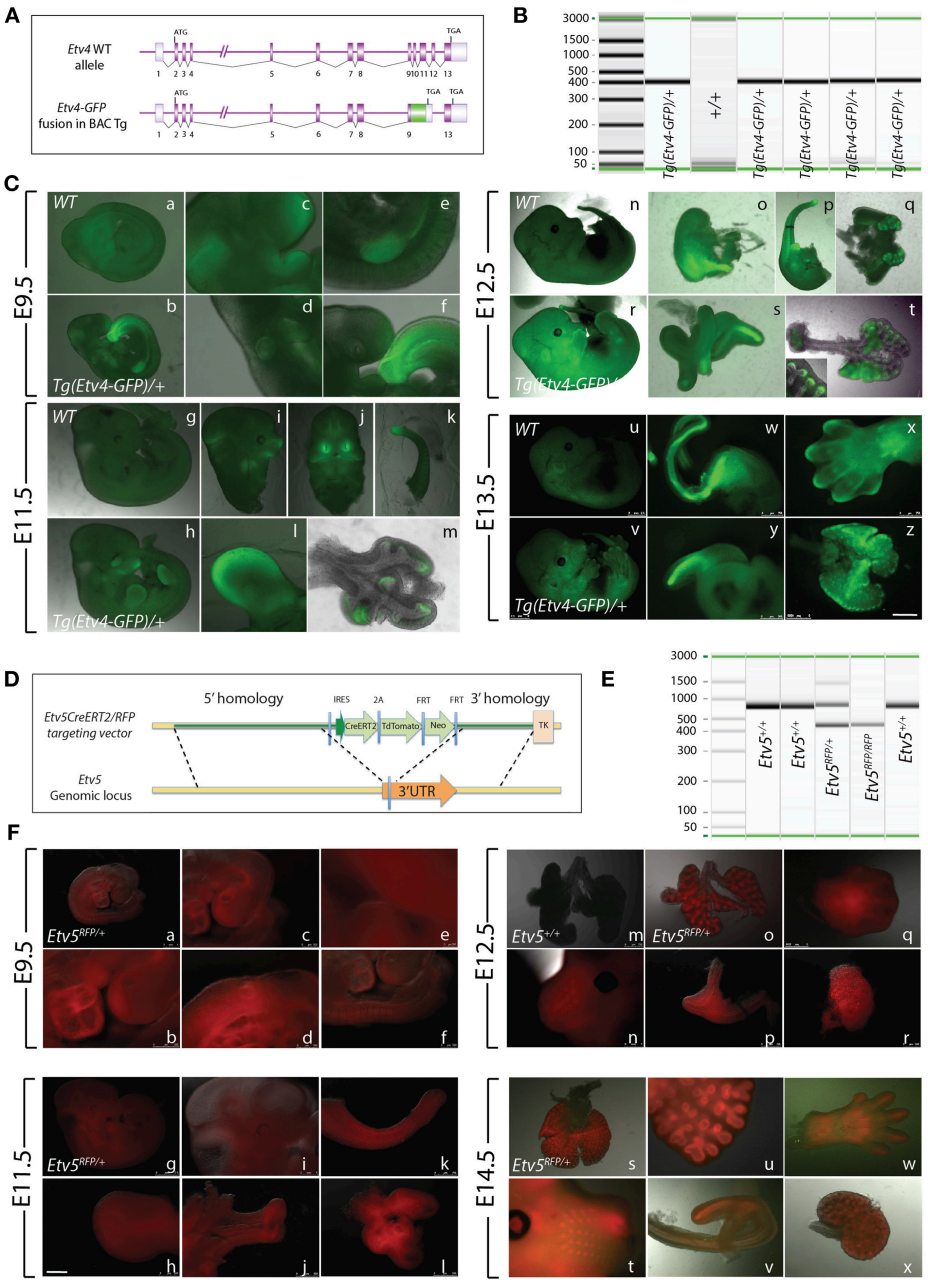
Early embryonic expression of ETV4-GFP and ETV5-RFP **(A)** *Tg(Etv4-GFP)* genetic construct. **(B)** Genotype detecting the *Tg(Etv4-GFP)* transgene at ~400 bp. **(C)** ETV4-GFP expression pattern in various embryonic organs at E9.5 (a–f), E11.5 (g–m), E12.5 (n–t), and E13.5 (u–z). See text for details. Note the increased expression of ETV4-GFP in the distal tips of the lung (see inset in “t,” and “z”). Scale bar: (a,b,g,h,n,r,u,v) 2.5 mm; (c–f,i–l,o–q,s,y) 500 μm; (w,x) 750 μm; (t,m,z) 1,000 μm. **(D)** *Etv5*^*RFP*^ genetic construct. **(E)** Genotype detecting the *Etv5*^*RFP*^ transgene at ~450 bp and the wild type *Etv5* at ~827 bp. **(F)** ETV5-RFP expression pattern in various embryonic organs at E9.5 (a–f), E11.5 (g–l), E12.5 (o–r), and E14.5 (s–x). Please note that the *Etv5*^+/+^ lungs do not show any autofluorescence (Figure 1Fm). See text for details. Note the differential expression level of ETV5-RFP in the distal lung epithelium vs. the proximal conducting airways (see “o” and “s”). Scale bar: (a,i,n,t) 1 mm; (b) 250 μm; (c,d,f,h,j,l,m,o,q-s,u,w,x) 500 μm; (e) 100 μm; (g) 2.5 mm; (k,p,v) 750 μm.

The *Tg(Etv4-GFP)* mouse line was created by Lamballe et al. (2011). These researchers used a bacterial artificial chromosome containing the *Etv4* gene fused with a green fluorescent protein gene (*GFP*) in frame with exon 9, to generate the *Tg(Etv4-GFP)* transgenic mouse line (Figure 1A). In the process, exons 10–12 of the *Etv4* construct were deleted. Thus, the randomly inserted *Tg(Etv4-GFP)* transgene is non-functional, and does not interfere with endogenous *Etv4* activity.

To characterize *Tg(Etv4-GFP)* expression in various organs during development, embryos were generated at different stages (E9.5, E11.5, E12.5, and E13.5). *Tg(Etv4-GFP)* was crossed with wild type mice to generate controls (*Etv4*^+/+^) and transgenic embryos (*Tg(Etv4-GFP)/*+) at different developmental stages. Figure 1B shows the PCR-based strategy designed to detect the band corresponding to the presence of the transgene (see sequence of the primers in materials and methods). Figure 1C shows embryos negative for GFP (Figures 1Ca,g,n,u) and positive for GFP (Figures 1Cb,r,h,v) at different developmental stages. At E9.5, GFP was detected in the nasal placode, the mandibullary and maxillary processes, and at the mid-brain/hindbrain junction (Figures 1Cc), the anterior limb bud and the mammary line (Figures 1Ce), the otic placode (Figures 1Cd), and the tail bud (Figures 1Cf). Similar expression sites were found at E11.5 (Figures 1Ch–l). Close examination of the lung indicated that GFP was expressed specifically at the tips in the epithelium and mesenchyme (Figures 1Cm). This expression overlaps with the previously reported endogenous *Etv4* expression in the E12.5 lung (Yin et al., 2011). At E12.5, the external expression of GFP did not appear as specific. However, upon dissection of internal organs, GFP was enriched in the posterior (glandular) stomach (Figures 1Co), the developing kidneys (Figures 1Cq), the epithelium of the cecum (Figures 1Cs), and the lung epithelium and mesenchyme (Figures 1Ct, see also insert in figure 1t). At E13.5, GFP was found in similar places (Figures 1Cw,y,z). In addition, GFP was located at the tip of the forming digits (Figures 1Cx).

### *Etv5^*CreERT*2−*RFP*/+^* Expression During Early Embryonic Development

The *Etv5*^*CreERT*2−*RFP*/+^ mouse line was recently generated by Barry Stripp (Cedars-Sinai Medical Center, Los Angeles, USA). In this line, a knock-in of *CreERT2/RFP* in the 3′ untranslated region of the *Etv5* gene was carried in embryonic stem cells (Figure 1D). Recombinant clones were used to generate *Etv5*^*CreERT*2−*RFP*/+^ knock-in mice. Figure 1E shows the PCR based strategy to detect the presence of the *Etv5*^*RFP*^ knock in allele in heterozygous or homozygous embryos (see sequence of the primers in materials and methods). We performed a similar characterization on this line as we did on the *Tg(Etv4-GFP)* mouse line.

*Etv5*^*CreERT*2−*RFP*/+^ mice were crossed with wild type mice to generate controls (*Etv5*^+/+^) and heterozygous embryos (*Etv5*^*CreERT*2−*RFP*/+^) at different developmental stages. During the E9.5 and E11.5 stages, RFP expression was general throughout the embryo (Figures 1Fa,g). At E9.5, expression was seen in the heart (Figures 1Fb), otic placode (Figures 1Fd), and nasal placode (Figures 1Fc). Expression in the tail was seen at E9.5 and E11.5 (Figures 1Ff,k), and in limb buds, limbs and the tips of forming digits in all stages (Figures 1Ff,h,q,w). Expression was seen in whisker regions (Figures 1Fn,t). After dissection, RFP was found to be enriched in the anterior stomach (Figures 1Fp), kidneys (Figures 1Frx), and epithelium of the cecum (Figures 1Fj,v). The expression of RFP in the lung at E11.5 did not appear enriched in the epithelium (Figures 1Fl). However, in later stages, RFP expression did appear enriched in the epithelium (Figures 1Fo,s,u), not only distally, but throughout the epithelium, with the expression disappearing in the main bronchi and trachea (e.g., Figures 1Fo,s, see also **Figures 4**, **6**).

### Expression of *Etv4* and *Etv5* During Embryonic Lung Development

**Figure 2:**
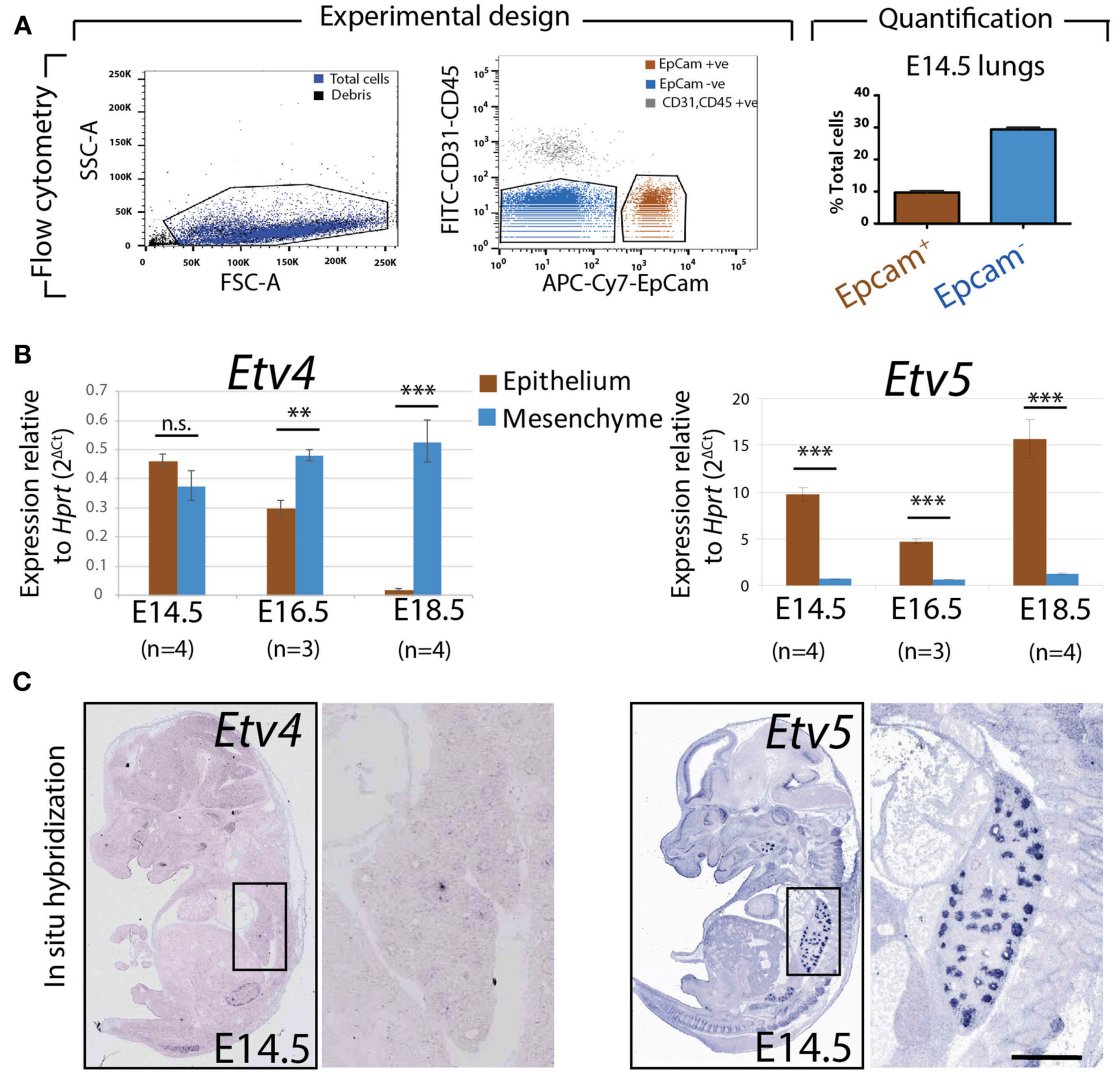
Expression of *Etv4* and *Etv5* in isolated embryonic lung epithelium and mesenchyme **(A)** Experimental design for the FACS-based isolation of embryonic lung epithelial and mesenchymal cells at E14.5 (*n* = 4; 44.88 ± 17.54 ng/μl RNA from epithelial cells, 64.70 ± 18.10 ng/μl RNA from mesenchymal cells), E16.5 (*n* = 3; 16.38 ± 5.69 ng/μl RNA from epithelial cells, 75.18 ± 13.79 ng/μl RNA from mesenchymal cells), and E18.5 (*n* = 4; 28.77 ± 3.06 ng/μl RNA from epithelial cells, 44.89 ± 15.30 ng/μl RNA from mesenchymal cells). Approximately 10 and 30% of the cells in E14.5 lungs were epithelial and mesenchymal, respectively. **(B)** RT-qPCR for *Etv4* and *Etv5* in isolated epithelial and mesenchymal cells. Note the different scales of the y-axis between *Etv4* and *Etv5* graphs. (Data are presented as geometric mean ± SE; n.s. = not significant, ^**^p < 0.01, ^***^p < 0.001). **(C)** *In situ* hybridization from the genepaint database showing the expression of *Etv4* and *Etv5* in E14.5 embryos.

To assess the relative expression of *Etv4* and *Etv5* during embryonic lung development, epithelial and mesenchymal cells from embryonic wild type mouse lungs were separated using FACS at different stages (E14.5, E16.5, and E18.5). Please note that while our protocol allows excluding the CD31-positive endothelium and CD45-positive hematopoietic cells, the isolated mesenchyme (negative for the surface marker Epcam, an epithelial cell adhesion molecule used to isolate epithelial cells) was a mixture of nerve cells, smooth muscle cells, mesothelial cells and resident mesenchymal cells. RNA was extracted from the Epcam-positive (epithelial) and Epcam-negative (mesenchymal) cells and qPCR analysis was performed (Figure 2A). *Etv4* expression was seen in both the mesenchyme and epithelium during embryonic development. At E14.5, *Etv4* was more highly expressed in the epithelium than in the mesenchyme. However, at later stages (E16.5 and E18.5), *Etv4* expression decreased in the epithelium while remaining relatively constant in the mesenchyme (Figure 2B).

*Etv5* was likewise expressed in both the mesenchymal and epithelial compartments throughout embryonic development. However, its expression in the epithelium was much higher than in the mesenchyme (around 10 times higher at E14.5, and 15 times higher at E18.5). *Etv5* epithelial expression decreased at E16.5 before greatly increasing at E18.5, compared to E14.5 (Figure 2B).

The expression of *Etv4* and *Etv5* was also validated using the publicly available genepaint.org data base. While *Etv4* expression was diffusely seen in the lung epithelium and surrounding mesenchyme, *Etv5* showed very strong expression restricted to the epithelium (compare insets in Figure 2C).

### Dynamic GFP Expression Reports FGF10 Signaling

**Figure 3:**
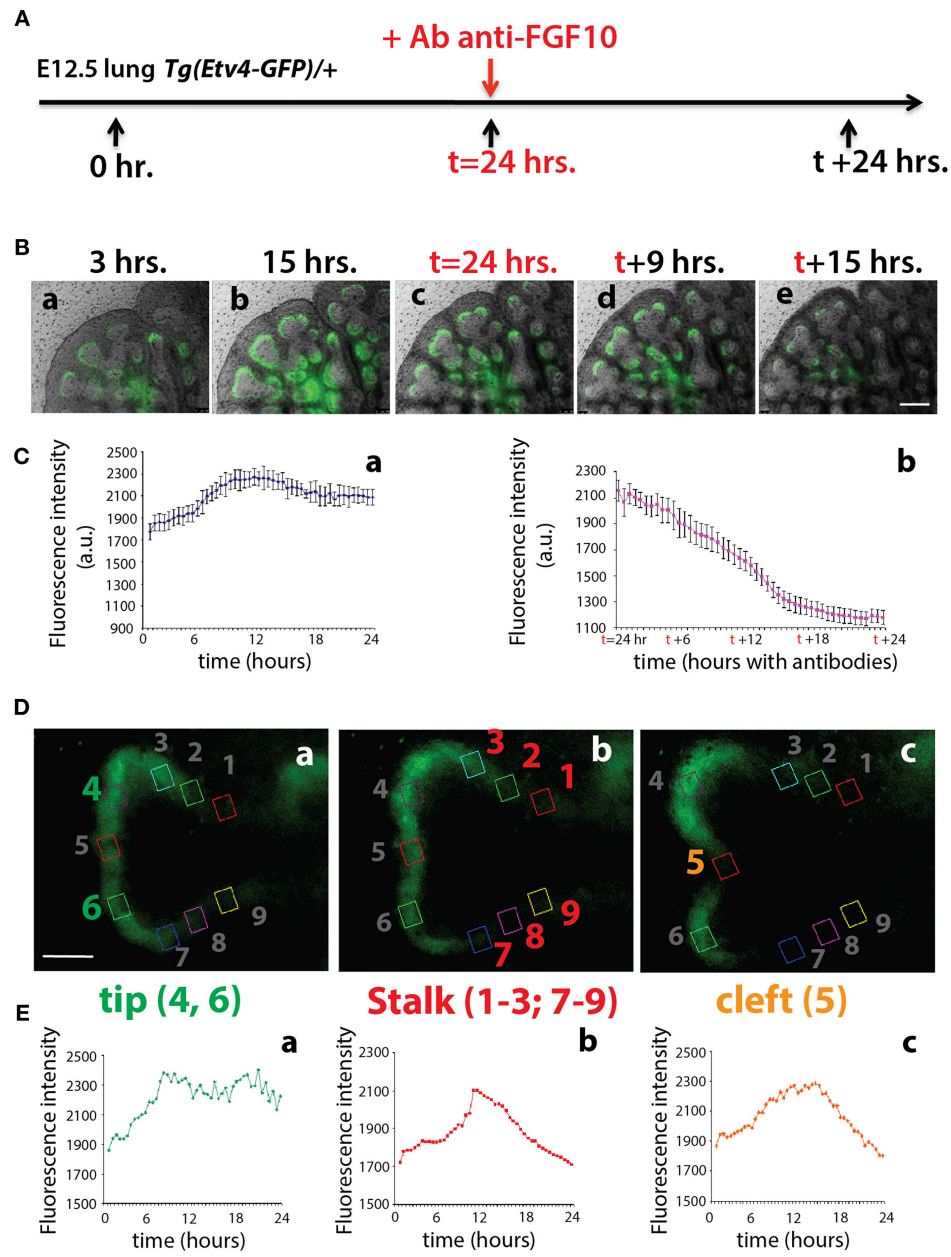
ETV4-GFP is dynamically expressed in regions of active FGF10 signaling during early lung development **(A)** Experimental design: E12.5 *Tg(Etv4-GFP)* lungs were isolated, cultured and live imaged for 48 h. After 24 h anti-FGF10 antibody was added to inhibit FGF10 activity. **(B)** Still images from different time points during the live imaging experiment. Note how ETV4-GFP expression increases before leveling off within the first 24 h (a–c), and, once the antibody is added, the expression decreases to almost zero by the end of the experiment (c–e). Scale bar: 125 μm. **(C)** Global ETV4-GFP fluorescence intensity measured at various time points before (a) and after (b) adding the FGF10 blocking antibody. (*n* = 3; data are presented as average fluorescence intensity in arbitrary units (a.u.) ± standard deviation). **(D)** Example images of a branching tip at three successive time points (a–c), highlighting three regions of dynamic ETV4-GFP expression: the tip (4 and 6), the stalk (1–3, and 7–9), and the cleft (5). See text for details. Scale bar: 30 μm. **(E)** Representative plot of ETV4-GFP expression in three independent regions [(a) tip, (b) stalk, and (c) cleft] of a single bud over a period of 24 h (*n* = 1; a.u. = arbitrary units).

To monitor the expression of *Tg(Etv4-GFP)* in the epithelial buds of pseudoglandular stage lungs in the context of FGF10 signaling, E12.5 *Tg(Etv4-GFP)* lungs were cultured and live imaged for 24 h, after which FGF10 signaling was blocked using an anti-FGF10 antibody for an additional 24 h (Figure 3A). During the first 24 h, ETV4-GFP was dynamically expressed, showing greater expression at the distal tips of growing buds, in likely regions of active FGF10 signaling (see Figures 3Ba–c and Supplementary Movie 1). During the FGF10 inhibition, ETV4-GFP expression was greatly reduced. Still images from multiple time points during the live imaging from three independent lungs were used to quantify these global changes in fluorescence intensity (Figure 3C). We also confirmed that the loss of GFP expression of the E12.5 lungs after 24 h in culture was neither due to bleaching, nor to a normal decrease in ETV4-GFP expression. Supplementary Figure 1 shows that the expression of ETV4-GFP in E12.5 transgenic lungs was maintained in culture for at least 38 h. Furthermore, still images of individual buds were used to quantify the dynamic expression of ETV4-GFP in three regions of the bud during a branching event (Figures 3D,E). Intensity was measured during new branch formation at the tip (regions 4 and 6), stalk (regions 1–3; 7–9) and cleft (region 5). Tip ETV4-GFP fluorescence intensity initially increased before stabilizing (Figures 3Ea), whereas stalk and cleft fluorescence intensity increased before decreasing to initial levels (Figures 3Eb,c). The expression patterns of the stalk and cleft reflected the initial single bud branching into two buds.

These results suggest that the *Tg(Etv4-GFP)* mouse line can be used as a valid tool to report FGF10 signaling in the distal tips of lungs during pseudoglandular development. This conclusion is supported by the previously described dynamic expression pattern of *Sprouty2* during the branching process (Mailleux et al., 2001). *Sprouty2* is a well-accepted target of FGF10 signaling in the lung, and displays a remarkably similar expression pattern to what we found for *Tg(Etv4-GFP)*.

### Quantification of Dynamic ETV5-RFP Fluorescence

**Figure 4:**
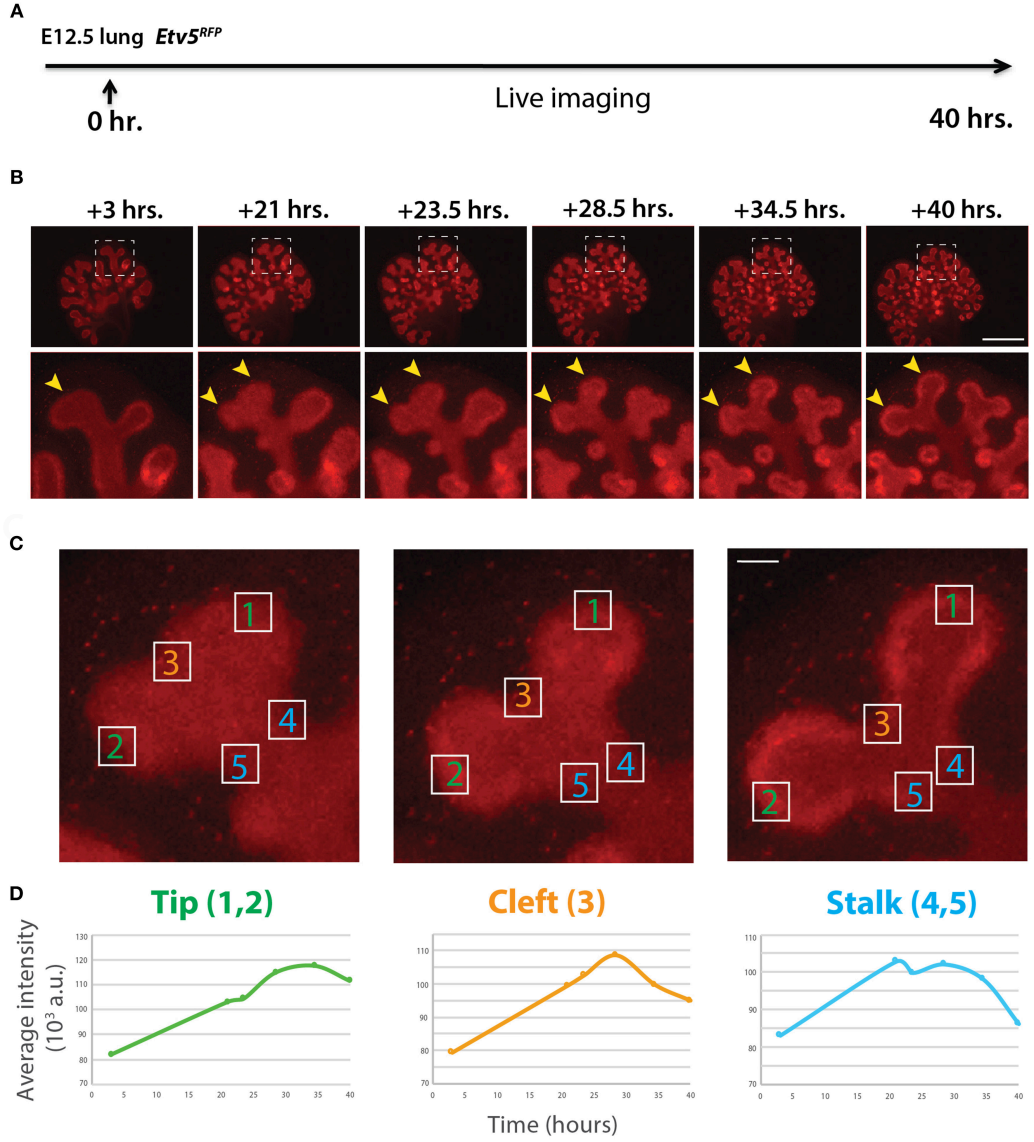
Expression pattern of ETV5-RFP in distal lung epithelium during early development **(A)** Experimental design: E12.5 *Etv5*^*RFP*^ lung explants were cultured for 40 h and live imaged. **(B)** Still images showing representative ETV5-RFP expression both globally and in the distal tips (boxes). Arrows indicate increased expression in the tips over time. Scale bar: (Top row) 500 μm; (Bottom row) 125 μm. **(C)** Example images of a branching tip at three successive time points (a–c), highlighting three regions of dynamic ETV5-RFP expression: the tip (1 and 2), the cleft (3), and the stalk (4 and 5). See text for details. Scale bar: 37 μm. **(D)** Representative plot of ETV5-RFP expression in three independent regions [(a) tip, (b) stalk and (c) cleft] of a single bud over a period of 40 h (*n* = 1; a.u. = arbitrary units).

Similar to the analysis of ETV4-GFP expression in early lung development, the expression of ETV5-RFP in branching epithelial buds was assessed. E12.5 *Etv5*^*CreERT*2−*RFP*/+^ lungs were cultured and live imaged for 40 h (Figure 4A). During this time ETV5-RFP was expressed throughout the epithelial tree at the exception of the primary bronchi and trachea. This was in contrast with the expression of ETV4-GFP, which was only at the tip. Interestingly, the more proximal regions of ETV5-RFP expression were not localized to known areas of FGF10 signaling. Close-up examination of ETV5-RFP indicated higher expression in distal tips compared to more proximal regions (see arrows in Figure 4B and Supplementary Movie 2). Finally, we quantified the fluorescence intensity of bud regions during a branching event using still images at multiple time points (Figures 4Ca–c). Fluorescence intensity at the tip (regions 1 and 2) increased before leveling off around 30 h, and then decreased slightly (Figures 4Da), whereas intensity at the cleft and stalk regions (regions 3 and 4–5, respectively) initially increased before decreasing to original levels (Figures 4Db,c). This temporal expression pattern was similar to what was found for *Tg(Etv4-GFP)* lungs, and is likely a consequence of the initial bud branching into two buds.

### Model to Inhibit FGF10 Confirms *Etv4* and *Etv5* Are Downstream of FGF10/FGFR2b Signaling

**Figure 5:**
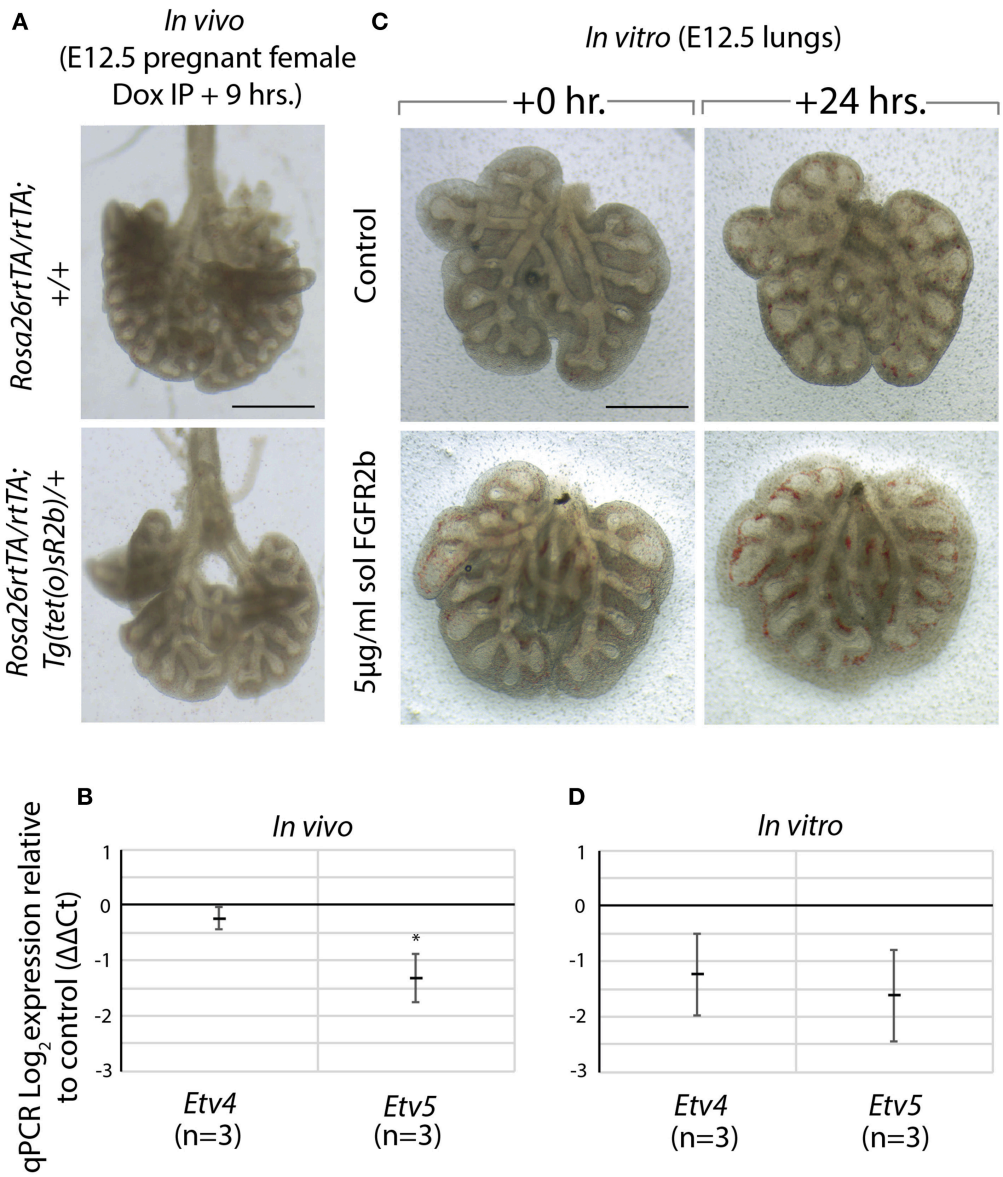
Model to inhibit FGF10 *in vivo* and *in vitro* demonstrates *Etv4* and *Etv5* are downstream of FGF10/FGFR2b signaling **(A)** *In vivo* model to inhibit FGF10/FGFR2b signaling: pregnant females carrying experimental (*Rosa26*^*rtTA*/*rtTA*^*; Tg*^*Tet*(*o*)*sR*2*b*/+^) and littermate control (*Rosa26*^*rtTA*/*rtTA*^*; Tg*^+/+^) embryos were injected with doxycycline. Embryonic lungs were isolated 9 h later. *Scale bar:* 500 μm. **(B)** Corresponding RT-qPCR analysis showing *Etv4* and *Etv5* expression in experimental vs. control lungs. **(C)** *In vitro* model to inhibit FGF10/FGFR2b signaling: E12.5 lung explants were cultured for 24 h with (experimental) or without (control) 5 μg/ml recombinant (soluble) FGFR2b. Scale bar: 500 μm. **(D)** Corresponding RT-qPCR analysis showing *Etv4* and *Etv5* expression in experimental vs. control lungs. (Data are presented as mean ± SEM; ^*^p < 0.05).

We made use of a model to inhibit all FGFR2b ligands *in vivo* via inducible expression of a dominant negative form of the FGFR2b receptor, called soluble FGFR2b (*Rosa26*^*rtTA*/*rtTA*^*; Tg(tet(o)sR2b)/*+) (Parsa et al., 2008, 2010). Upon administration of doxycycline, soluble FGFR2b is produced and secreted from cells, functionally inhibiting all FGFR2b ligands from properly signaling. We have previously shown that at E12.5, FGF10 is the predominant FGF ligand signaling in the lung (Bellusci et al., 1997b). Therefore, induction of soluble FGFR2b at this time point inhibits FGF10 signaling. As Figure 5A shows, after only 9 h of FGF10 inhibition, experimental lungs were smaller, displayed simplified branching, and had fewer distal buds. Furthermore, the expressions of *Etv4* and *Etv5*, as evaluated by RT-qPCR, were reduced (Figure 5B).

A similar approach to inhibiting FGFR2b signaling was conducted *in vitro*. In this experiment, wild type E12.5 lungs were harvested and cultured with (experimental) or without (control) recombinant FGFR2b added to the culture medium for 24 h (Figure 5C). A similar phenotype to that seen *in vivo* was produced in experimental lungs, while the downregulation of *Etv4* and *Etv5* mRNA was even more pronounced (Figure 5D).

Taken together, these results suggest that FGF10 signaling can be inhibited both *in vitro* and *in vivo*, and that *Etv4* and *Etv5* are regulated by FGF10.

### ETV5-RFP Expression Reports FGF10 Signaling *in vitro*

**Figure 6:**
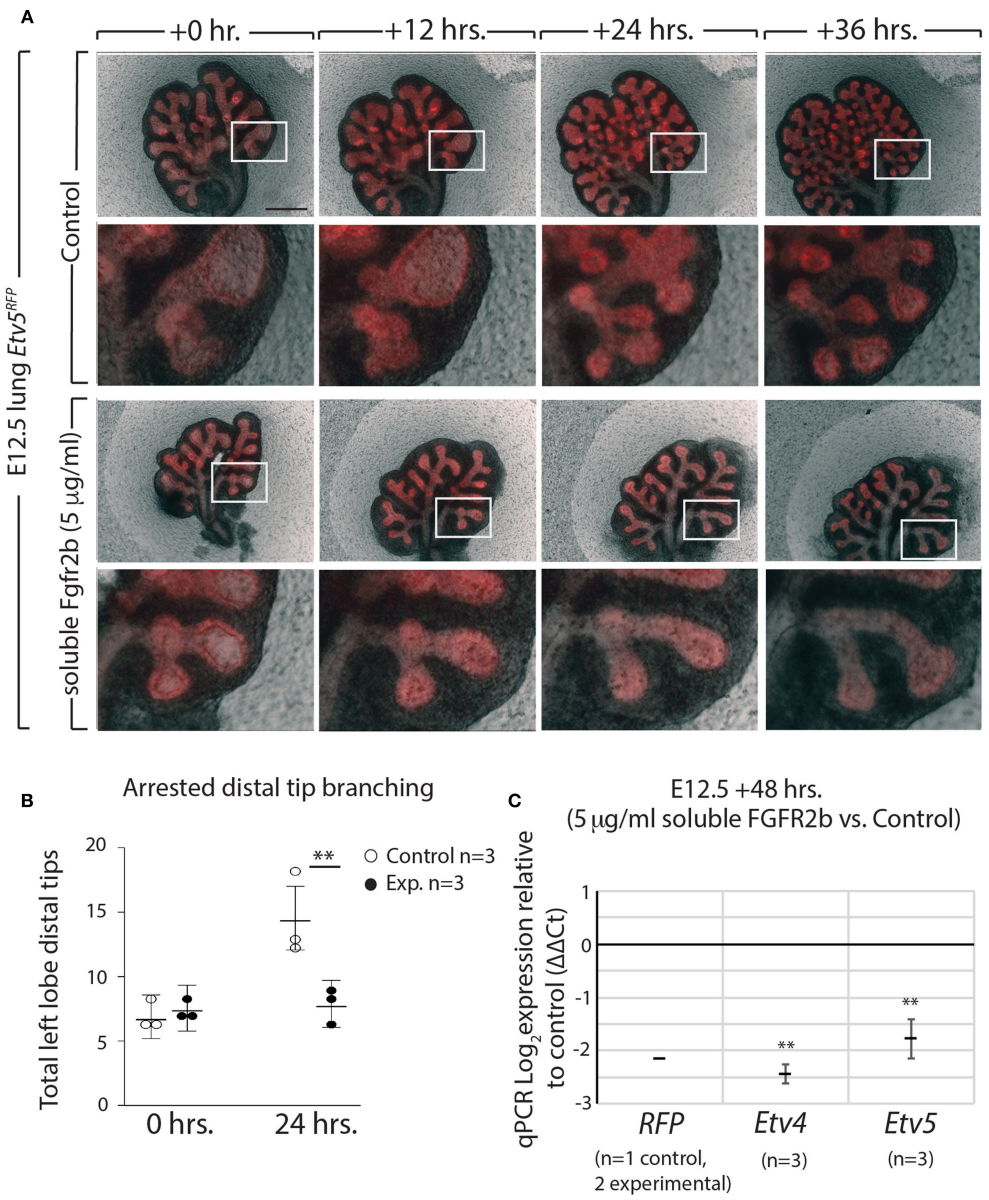
ETV5-RFP reports FGF10/FGFR2b signaling *in vitro* **(A)** E12.5 *Etv5*^*RFP*^ lungs were harvested and cultured with (experimental) or without (control) 5 μg/ml recombinant (soluble) FGFR2b for 48 h. *Scale bar:* (Top rows) 500 μm; (Boxes) 125 μm. **(B)** Left lobe distal tips show arrested branching in experimental lungs after 24 h. FGF10/FGFR2b inhibition, compared to control lungs. While both control lungs and experimental lungs had a similar number of average tips at 0 h, only control lungs showed an increase in branching over time (Data are presented as mean with 95% CI; ^**^p < 0.01). **(C)** RT-qPCR analysis of the lungs showing downregulation of *RFP, Etv4*, and *Etv5*, in experimental lungs, demonstrating the usefulness of the *Etv5*^*RFP*^ line to report FGF10/FGFR2b signaling. Note, we only had one control *Etv5*^*RFP*^ lung and two experimental *Etv5*^*RFP*^ lungs, thus statistical analysis was not possible (Data are presented as mean ± SEM; ^**^p < 0.01).

Using our *in vitro* approach to inhibit FGF10/FGFR2b signaling in pseudoglandular stage lungs, we investigated whether the *Etv5*^*CreERT*2−*RFP*^line could be used as a tool to report FGF10/FGFR2b signaling. E12.5 *Etv5*^*CreERT*2−*RFP*^ lungs were cultured and live imaged for 48 h with (experimental) or without (control) recombinant soluble FGFR2b (Figure 6A). As can be seen in the still images, the soluble FGFR2b treated lungs showed arrest in branching over time, compared to the control (Figure 6B). This phenotype is a hallmark of inhibited FGF10 signaling. Furthermore, demonstrating the usefulness of this line to report FGF10/FGFR2b signaling, the fluorescence intensity of the ETV5-RFP decreased over time in experimental lungs, while that of the controls remained constant. This finding is supported by RT-qPCR results after 48 h, which show a decrease in the expression of *RFP, Etv4*, and *Etv5* in experimental lungs compared to controls (Figure 6C).

These results not only confirm that soluble FGFR2b can be used to inhibit FGF10 signaling in *Etv5*^*CreERT*2−*RFP*^ lung explants, but they also suggest that the *Etv5*^*CreERT*2−*RFP*^ line responds to FGF10/FGFR2b signaling in the developing lung.

## Conclusion

In conclusion, the *Tg(Etv4-GFP)* and *Etv5*^*RFP*^ reporter mouse lines appear to be promising tools to monitor FGF10/FGFR2b signaling in early lung development. These tools will have to be further validated at later stages and in other organs of interest.

## Ethics Statement

Animal experiments, harvesting organs and tissues from wild type and mutant mice following euthanasia using pentobarbital was approved at Justus Liebig University Giessen by the federal authorities for animal research of the Regierungspraesidium Giessen, Hessen, Germany (Approved Protocol GI 20/10 Nr. G 84/2016).

## Author Contributions

SB, CC, C-MC, BS, and FH concept and design. MJ, AL, SD, and AS acquisition of data. MJ, SB, C-MC, and SD analysis and interpretation. MJ and SB drafting and editing of the manuscript. All authors read and approved the final manuscript.

### Conflict of Interest Statement

The authors declare that the research was conducted in the absence of any commercial or financial relationships that could be construed as a potential conflict of interest.

## Supplementary Material

Supplementary Movie 1 ETV4-GFP in the E12.5 developing lung for 24 h.

Supplementary Movie 2 ETV5-RFP in the E12.5 developing lung for 24 h.

Supplementary Figure 1 ETV4-GFP expression is stable for at least 38 h *in vitro* To monitor the expression pattern of ETV4-GFP in E12.5 lungs *in vitro*, lungs were cultured and live imaged in control medium **(a–d)**, with additional recombinant FGF7 **(e–h)**, or additional recombinant FGF10 **(i–l)** for 38 h. Scale bar: 125 μm.
