# Supplementary figures and images for: Characterization of Tg(Etv4-GFP) and Etv5RFP Reporter Lines in the Context of Fibroblast Growth Factor 10 Signaling During Mouse Embryonic Lung Development
Source: Front Genet. 2019 Mar 14;10:178. doi: 10.3389/fgene.2019.00178 (PMC6426760; doi:10.3389/fgene.2019.00178)

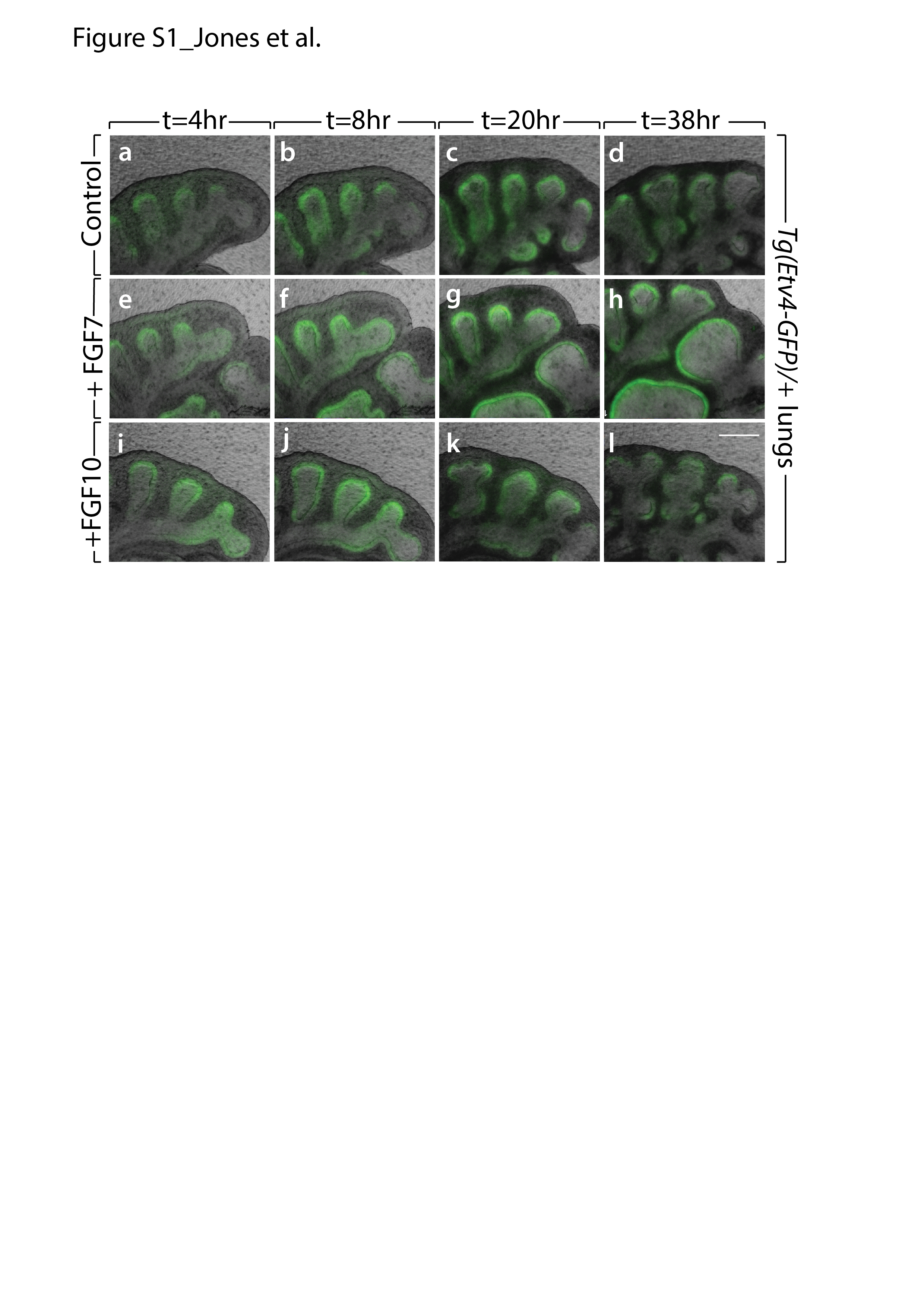

Supplement: Supplementary Figure 1 — ETV4-GFP expression is stable for at least 38 h in vitro To monitor the expression pattern of ETV4-GFP in E12.5 lungs in vitro, lungs were cultured and live imaged in control medium (a–d), with additional recombinant FGF7 (e–h), or additional recombinant FGF10 (i–l) for 38 h. Scale bar: 125 μm. [file Image_1.JPEG]
